# Implications of HIV-1 Nef for “Shock and Kill” Strategies to Eliminate Latent Viral Reservoirs

**DOI:** 10.3390/v10120677

**Published:** 2018-11-30

**Authors:** Xiaomei T. Kuang, Mark A. Brockman

**Affiliations:** 1 Department of Molecular Biology and Biochemistry, Simon Fraser University, Burnaby, BC V5A 1S6, Canada; xtk@sfu.ca; 2 Faculty of Health Sciences, Simon Fraser University, Burnaby, BC V5A 1S6, Canada; 3 British Columbia Centre for Excellence in HIV/AIDS, Vancouver, BC V6Z 1Y6, Canada

**Keywords:** HIV-1, Nef, viral latency, shock and kill

## Abstract

Finding a cure for HIV is challenging because the virus is able to integrate itself into the host cell genome and establish a silent state, called latency, allowing it to evade antiviral drugs and the immune system. Various “shock and kill” strategies are being explored in attempts to eliminate latent HIV reservoirs. The goal of these approaches is to reactivate latent viruses (“shock”), thereby exposing them to clearance by viral cytopathic effects or immune-mediated responses (“kill”). To date, there has been limited clinical success using these methods. In this review, we highlight various functions of the HIV accessory protein Nef and discuss their double-edged effects that may contribute to the limited effectiveness of current “shock and kill” methods to eradicate latent HIV reservoirs in treated individuals.

## 1. Introduction

The presence of long-lived latent HIV reservoirs is the major hurdle to achieving combination antiretroviral therapy (cART)-free viral remission and a potential cure. To date, the only case of an apparently successful HIV cure is the “Berlin patient”, who received two hematopoietic stem cell transplants from separate CCR5∆32 homozygous donors to treat his leukemia [1,2]. He displays no evidence of HIV infection despite remaining off therapy since 2007. Such transplants are exceptionally high-risk procedures and are thus not applicable to the global population of approximately 37 million HIV-infected individuals [3]. Furthermore, subsequent attempts to use similar transplantation strategies in HIV-infected individuals who were also undergoing cancer therapy have been unsuccessful, with viral rebound observed within weeks to months following cART discontinuation [4]. Therefore, the development of safer and more effective methods to reduce or eliminate latent HIV reservoirs in cART-treated individuals is a high priority for researchers and the community.

Different potentially curative approaches for HIV are currently under development, ranging from pharmacological approaches to immune-based and genetic therapies. Of these, the most intensively investigated strategies are the “shock and kill” methods to reduce or eliminate replication-competent latent HIV reservoirs in cART-treated individuals [5]. However, this strategy requires the induction of viral protein expression, including the regulatory and accessory proteins Tat, Rev, Nef, Vif, Vpr and Vpu, which could interfere with this process. In this article, we introduce the “shock and kill” method, describe the multi-functional viral accessory protein Nef, and consider how Nef may alter the efficiency of HIV cure approaches by modulating the viral reactivation from latency or the subsequent elimination by host immune mechanisms.

## 2. “Shock and Kill” Method

**Figure 1:**
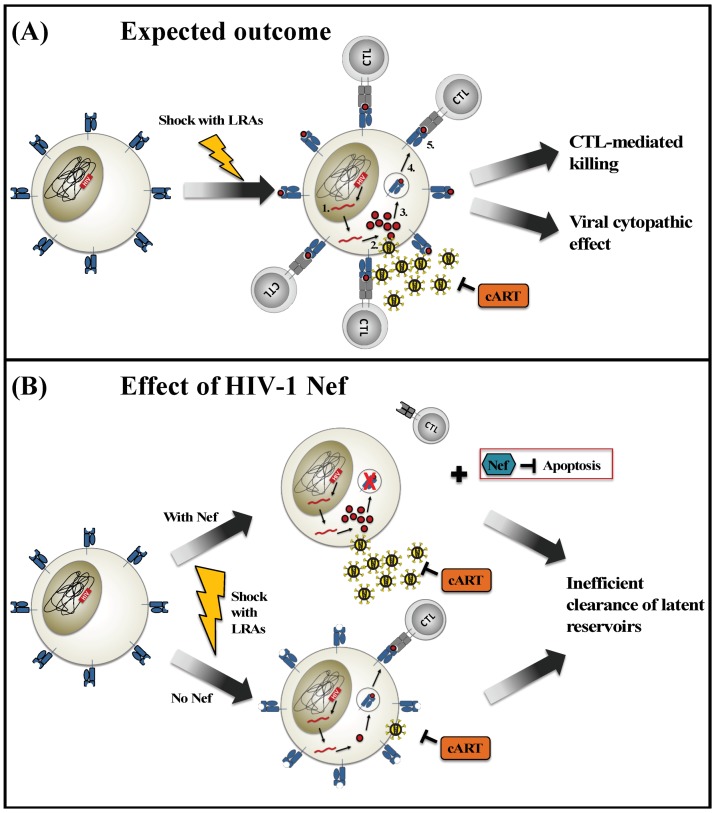
Impact of Nef on “shock and kill” methods to eradicate HIV reservoirs. (**A**) This illustration displays the expected outcome of a latent HIV-infected T cell following induction with latency-reversing agents (LRAs) (“shock”) in the presence of combination antiretroviral therapy (cART). The integrated HIV proviral genome is transcribed (1) and translated into viral proteins (2). Some viral proteins are degraded into peptide antigens and loaded onto HLA class I molecules (3) for presentation at the cell surface (4). The recognition of peptide-HLA complexes by cytotoxic T lymphocytes (CTL) (5) induces cytolytic mechanisms that kill the virus-infected cell. Alternatively, the expression of viral proteins may induce viral cytopathic effects that result in the death of the infected cell. (**B**) This illustration displays the potential contributions of the viral Nef protein to modulate the reactivation and elimination of latent HIV-infected cells by “shock and kill” methods. In the presence of Nef, viral protein expression is robust, but HLA class I molecules are down-regulated from the cell surface and cellular apoptosis is inhibited. In the absence of Nef, viral protein expression is reduced, thus limiting the amount of viral antigen that is available for presentation on HLA class I. In both scenarios, CTL-mediated recognition and elimination of newly reactivated HIV-infected cells may be hindered.

An illustration of the “shock and kill” method to eliminate latent HIV-infected cells in cART-suppressed individuals is shown in Figure 1A. Using latency-reversing agents (LRAs) that modulate cellular chromatin structure or otherwise stimulate the HIV 5’ LTR promoter, viral gene transcription is reactivated (“shock”) in latent HIV-infected cells. The subsequent viral protein expression, followed by the proteasomal processing and presentation of viral antigens on the cell surface in complex with human leukocyte antigen class I (HLA-I) molecules is then expected to result in the elimination (“kill”) of these cells by cytotoxic T lymphocytes (CTL). Alternatively, reactivated cells may undergo apoptosis due to the accumulation of viral cytopathic effects (CPE). By maintaining individuals on cART treatment during this process, viral replication and seeding of new HIV reservoirs is avoided.

### 2.1. Inefficient Viral Reactivation Using LRAs

Different classes of LRAs have been identified and tested for their ability to “shock” the latent HIV reservoir. In particular, pan-histone deacetylase inhibitors (HDACi), such as vorinostat [6], romidepsin [7], and panobinostat [8], are currently among the most promising classes of LRAs. Through the inhibition of multiple HDAC enzymes, HDACi increases the overall level of acetylation on histone molecules. This ultimately reduces chromatin condensation and promotes nonspecific increases in both host and viral gene expression. Many HDACi are FDA-approved for cancer treatment, and their pharmacological and toxicological profiles are known. Hence, HDACi have advanced quickly to human clinical trials in the context of HIV cure strategies, where they have demonstrated a range of abilities to induce latent viral reservoirs that broadly reflect their potency [9,10]. Several other classes of LRAs have also been tested in clinical studies. For example, disulfiram modestly reverses HIV latency by depleting PTEN (phosphatase and tensin homolog), which subsequently results in the activation of the PI3K/Akt pathway [11]. Protein kinase C (PKC) activators, such as prostratin and bryostatin, potently initiate HIV transcription in ex vivo experiments [12,13]; however, treatment with tolerable doses of bryostatin showed minimal ability to reactivate latent HIV in vivo in human studies [14]. Additional LRAs such as Toll-like receptor (TLR) agonists [15] and cytokines (i.e., interleukin-7 and -15) [16] are also being examined. Overall, none of these clinically relevant LRAs has been shown to reverse HIV latency potently in infected individuals. In fact, one ex vivo study indicated that many latent virus-infected cells remained uninduced despite strong T cell stimulation using phytohemagglutinin (PHA) or phorbol 12-myristate 13-acetate (PMA) plus ionomycin [17], suggesting that repeated induction using more potent LRAs may be necessary to achieve a clinically beneficial outcome.

### 2.2. Ineffective Clearance of Reactivated Cells

Despite some success with inducing latent HIV gene expression in cART-treated individuals, no significant reductions in viral reservoir size have been observed in vivo. This suggests that immune-mediated clearance of reactivated cells and/or viral CPE is inefficient. While it is often assumed that the production of HIV proteins such as Vif and Vpr could cause cell death due to viral CPE [18], Shan et al. demonstrated that the presence of viral protein expression was not associated with a spontaneous reduction of latent HIV-infected cells following reactivation using vorinostat [19]. In addition to the limited impact of viral CPE, the same study showed that CTL isolated from most cART-treated individuals were unable to eliminate latent cells reactivated ex vivo with HDACi efficiently without pre-stimulation using HIV antigens [19]. Nevertheless, a more recent study using Nef- and Gag-stimulated CTL was unsuccessful in eliminating reactivated cells and reducing the size of latent reservoirs [20]. The lack of CTL-mediated killing is potentially attributed to impaired CTL functionality and/or limited viral peptide presentation by reactivated cells. While there has been controversy regarding LRA-associated CTL impairment, results from clinical studies showed no evidence of CTL dysfunction in patients who were treated with HDACi [7,21]. Nonetheless, increasing evidence from in vitro studies are reporting associations between treatment with selected LRAs and CTL dysfunction. In particular, romidepsin, panobinostat, and vorinostat appeared to reduce the production of cytokines interferon-γ, tumor necrosis factor-α (TNF- α) and interleukin-2 [20,22]. Correspondingly, these HDACi-treated CTL displayed an impaired ability to eliminate HIV-infected cells [22]. On the other hand, limited studies have investigated HIV peptide presentation by reactivating cells. Clutton et al. observed impaired antigen presentation in reactivating cells due to inadvertent reduction in HLA class I expression following HDACi stimulation [23].

In summary, clinical studies have not reported a successful reduction of the latent viral reservoir in vivo [6,7,10,21]. The major hurdles encountered by these strategies include the inefficient induction of viral protein expression and the ineffective clearance of reactivated cells by the immune system.

## 3. Modulation of HIV-Infected Cells by Nef

HIV-1 Nef is a ~27 kDa myristoylated protein. It is encoded by the highly variable *nef* gene, which is located near the 3’ end of the viral genome. Nef is one of the earliest and most abundant viral proteins expressed by cells following infection [24,25,26,27], and presumably, following viral reactivation. Although Nef is often not required for HIV replication in vitro, it has been shown to be crucial for viral pathogenesis in vivo. Nef does not display any enzymatic activity; rather, it serves as a multi-functional adaptor protein that interacts with host proteins to interfere with a variety of processes in infected cells [28,29].

Nef downregulates CD4 expression on the surface of virus-infected cells [30] through clathrin-mediated endocytosis [31,32] and the increased endosomal retention [33,34] of CD4 molecules. Because CD4 is the primary receptor for HIV attachment and the entry into target cells, reduced CD4 expression allows a more efficient release of newly formed HIV particles [35,36], enhances virion infectivity [37] and inhibits superinfection [38]. Perhaps more important in the context of viral reactivation from latency, the interaction between CD4 and Env glycoproteins on the same cell has been shown to alter the conformation of Env to expose epitopes that are recognized by antibodies with potent antibody-dependent cellular cytotoxicity (ADCC) activity [39,40,41]. Hence, the efficient downregulation of CD4 by Nef can also protect infected cells from elimination by ADCC [42].

Nef is also well-known for its ability to evade the host immune response by selectively downregulating two HLA-I molecules, HLA-A and HLA-B [43,44,45]. This activity of Nef is genetically separable and mechanistically distinct from that of CD4 downregulation [46,47]. HLA-restricted CTL responses are associated with better control of viremia during primary HIV infection [48,49] and differential rates of clinical disease progression [50,51]. Thus, the reduced expression of HLA-A and HLA-B molecules on the surface of infected cells can protect them from CTL recognition and elimination [52]. In addition, the retention of HLA-C and HLA-E can inhibit the cytolytic activity of natural killer (NK) cells [44,45], preventing virus-infected cells from being eliminated through this innate immune mechanism.

A novel strategy to explain how Nef enhances viral infectivity was elucidated by two groups of researchers in 2015, who demonstrated that Nef can antagonize host restriction factors serine incorporator 3 and 5 (SERINC3/5) [53,54]. While understanding the precise mechanisms responsible for SERINC-mediated antiviral activity is currently an area of active investigation [55,56], the incorporation of SERINC3 or 5 into the membrane of newly formed virions significantly reduces their ability to form fusion pores with target cells, resulting in lower HIV infectivity [57]. To counteract these host restriction factors, Nef can downregulate SERINC3/5 from the surface of infected cells, which ultimately leads to the production of progeny virions that display higher infectivity [58].

Another critical role of Nef during HIV infection is its ability to modulate T cell signaling events. By downregulating CD4 and CD28 molecules on the surface of virus-infected T cells, Nef reduces the efficiency of T cell activation mediated through the T cell receptor (TCR) [30,59]. To further suppress the antigen-mediated stimulation of infected T cells, Nef binds Lck and redirects it to the trans-Golgi network (TGN), away from the plasma membrane where it can no longer participate in proximal TCR signal amplification events [60,61,62]. Together, the reduced availability of CD4, CD28 and Lck signaling molecules prevents the formation of an immunological synapse at the plasma membrane [60,61,63]. Paradoxically, while the altered trafficking of Lck interrupts TCR-mediated signaling at the plasma membrane, it permits the activation of Ras and downstream mitogen-activated protein kinase/extracellular signal-regulated kinases (MAPK/ERK) signaling events at the intracellular TGN compartment by forming a large complex that has been referred to as the Nef “signalosome” [62]. Alternatively, Nef can induce Ras activity via the formation of a Nef-associated kinase complex (NAKC), which is comprised of Nef, Lck, linker of activated T cells (LAT) and Ras proteins [62,64]. In synergy with activated Ras signaling, interaction between Nef and the endoplasmic reticulum-resident inositol triphosphate receptor (IP3R) can trigger calcium flux into the cytosol and induce TCR-independent activation of nuclear factor of activated T cells (NFAT) [65,66]. Together, Nef’s uncoupled effects on T cell activation pathways can simultaneously suppress activation-induced cell death (AICD) triggered by extracellular antigen recognition and also increase viral gene transcription.

Current evidence indicates that Nef may protect virus-infected cells from apoptosis, while simultaneously eliciting the death of bystander immune cells, which may enhance pathogenesis. To prevent infected cells from undergoing programmed cell death, Nef inhibits the activities of apoptosis signal-regulating kinase 1 (ASK1) [67], tumor suppressor p53 [68] and the pro-apoptotic protein Bcl-2-associated death promoter (BAD) [69]. In contrast, secreted Nef can upregulate Fas ligand induced apoptosis of uninfected bystander CD4^+^ T cells and CTL [70,71,72], thereby dampening the local immune response against HIV-infected cells. Transgenic mice expressing Nef display AIDS-like pathologies [73], raising the possibility that the induction of Nef by “shock and kill” methods may lead to toxicity, particularly in localized tissues that harbor latent viral reservoirs, such as lymph nodes or the central nervous system [74,75].

Finally, by manipulating cytoskeletal dynamics, Nef may promote a more permissive cellular environment to support viral replication or spread. Nef associates with the serine/threonine kinase p21 activated kinase 2 (PAK2) in a multiprotein complex and redirects its phosphorylation to a novel target, the actin depolymerization factor cofilin [76,77], which results in reduced F-actin turnover and actin cytoskeleton remodeling [78,79]. Consequently, this prevents F-actin accumulation at the immunological synapses upon TCR engagement [61], thereby contributing to the inhibition of AICD and prolonging the survival of infected cells [80].

## 4. The Double-Edged Effect of HIV-1 Nef

### 4.1. How Nef Might Enhance “Shock and Kill” Strategies

Many factors that promote HIV latency are likely to contribute to the inducibility of viral reservoirs upon treatment with an LRA. Even though Nef’s role in the context of latency is not fully characterized, several studies have highlighted its ability to induce viral reactivation. For example, Fujinaga et al. demonstrated that exogenous Nef activated virus production in latent cell lines (i.e., MOLT-20-2 and U1) as well as in peripheral blood mononuclear cells (PBMC) isolated from asymptomatic HIV-infected individuals [81]. Follow-up studies by the same group suggested that this effect was driven by Nef’s ability to induce Ras-mediated MAPK/ERK signaling [82]. The effect of Nef on latency reversal was confirmed in a separate study using U1 cells [83]. More recently, treatment using exogenous Nef alone was also found to be sufficient to activate the PI3K/Akt pathway and to increase HIV reactivation in the Jurkat-derived 1G5 latent T cell line [84].

In addition to Ras and Akt, Nef can also regulate the cellular activation status by interacting with other host proteins. Hence, it is not entirely surprising that Nef could activate latent HIV-infected cell lines. For instance, the presence of Nef can trigger the formation of NAKC and induce downstream Ras/MAPK activity [62,64]. Through its interaction with IP3R, Nef can trigger calcium flux into the cytosol and induce NFAT activation [65,66]. In both cases, early production of Nef during viral reactivation might enhance latent T cell activation. Moreover, previous studies reported that Nef can be released into the extracellular space either in soluble form [85,86] or within exosomes [87,88]. Both soluble and exosome-associated Nef have been shown to induce HIV reactivation in latently infected cells [81,89], but their proposed molecular mechanisms are distinct. In particular, soluble Nef may bind non-specifically to the surface of latent HIV-infected cells and be internalized via endocytosis [90,91]. After entering the cell, Nef can induce Ras/MAPK [82] and PI3K/Akt [84] signaling pathways that ultimately activate viral gene transcription. On the other hand, Nef increases the production of exosomes containing activated ADAM17 (a disintegrin and metalloprotease domain 17) [92], an enzyme that converts pro-TNF-α into its active form. The uptake of ADAM17-containing exosomes by target cells can induce the release of TNF-α [93], which subsequently binds to TNF receptor type 1 and activates NF-κB and c-Jun N-terminal kinase (JNK) pathways [94]. Additionally, Nef has been shown to increase exosome release, which presumably enhances the transfer of Nef-associated signaling activities to nearby cells [95]. Nef-mediated effects on cellular signaling are complex and their potential impacts on viral reactivation are not mutually exclusive. In fact, based on these previous findings, we speculate that Nef’s ability to enhance viral reactivation may be attributed to a positive feedback loop of cellular activation. Specifically, upon stimulation with LRAs, early Nef expression may increase viral gene expression. Subsequent secretion of soluble Nef and Nef/ADAM17-contaning exosomes could further increase the activation of latent cells through direct effects of Nef or TNF-mediated signaling pathways.

### 4.2. How Nef Might Impair “Shock and Kill” Strategies

Recent results by Huang et al. suggested that replication-competent latent proviruses may display resistance to elimination by HIV-specific CTL [96]. Hence, apart from LRA-associated impairments in CTL functions, the expression of Nef immediately following viral reactivation may further reduce the ability of CTL to recognize and eliminate latent reservoirs. Specifically, the ability of Nef to selectively downregulate surface HLA-I molecules [43,44,45] may allow reactivated cells to evade immune surveillance. In support of this theory, Mujib et al. used small molecules designed to inhibit Nef, which partially reversed HLA downregulation and promoted the elimination of reactivating cells by HIV-specific CTL [97]. While the ability of Nef to downregulate CD4 can prevent the ADCC-mediated elimination of productive virus-infected cells [42], no studies have examined this question in the context of latent viral reservoirs.

As the leading class of LRAs, HDACi triggers various apoptotic pathways to induce tumor cell death (reviewed in Reference [98]). While this strongly suggests that the use of certain LRAs could inadvertently induce apoptosis of latent reservoirs upon viral reactivation, the mechanism(s) involved have not been explored. Nonetheless, the ability of Nef to counteract multiple apoptotic pathways and promote cell survival could further hinder the clearance of reactivating reservoirs. First, Nef can bind directly to ASK-1 [67], an importance intermediate of Fas- and TNF-α-induced death signaling cascades [99,100], thereby preventing its dissociation from negative regulator thioredoxin [101]. Consequently, this inhibits the ASK-1-mediated activation of the downstream JNK signaling pathway to induce apoptosis [102]. Second, Nef can protect cells from undergoing p53-mediated apoptosis by binding and destabilizing p53, causing an overall reduction of this protein [68]. Third, the ability of Nef to associate with PI3K can induce downstream PAK-mediated phosphorylation of pro-apoptotic protein BAD [69]. Since phosphorylated BAD is incapable of forming heterodimers with anti-apoptotic proteins Bcl-2 and Bcl-X_L_, these proteins remain active for the promotion of cell survival [103].

Furthermore, broad reactivation of HIV proteins using LRAs may lead to AICD among the proportion of reservoir cells that is HIV-specific [104]. In this case, Nef’s ability to downregulate CD4 expression, modulate T cell signaling and cytoskeleton rearrangement may protect these cells from undergoing AICD. Taken together, early Nef expression following LRA-induced viral reactivation could inhibit CTL-mediated killing, apoptosis and AICD of latent reservoir, which may contribute to the lack of success seen using current “shock and kill” methods.

## 5. Conclusions

The efficiency of “shock and kill” strategies is determined by the degree to which latent HIV reservoirs are reactivated and subsequently eliminated in the host. We hypothesize that Nef might play a “dual” role in modulating both of these important factors (as illustrated in Figure 1B). While studies have demonstrated the use of exogeneous Nef to induce viral reactivation, Nef’s ability to mediate immune evasion and to enhance cell survival through the inhibition of apoptosis are also well documented. Nef leads to the downregulation of HLA-I molecules on the cell surface [43,44,45], which reduces the presentation of viral peptide antigens and impairs CTL-mediated recognition and cytolytic activity against reactivating reservoirs [52]. Additionally, Nef’s ability to modulate apoptotic pathways may prevent reactivated cells from dying due to viral cytopathic effects [67,69]. In contrast, latent cells that lack functional Nef may be unable to produce viral proteins efficiently. As a result, the presentation of viral peptides may be limited despite retaining high levels of HLA-I expression on the cell surface. Hence, the diverse roles played by Nef may create double-edged effects in the setting of a “shock and kill” strategy. Further studies to explore the possible impact of Nef and other viral accessory proteins, such as Vpr and Vpu, during HIV reactivation from latency may lead to enhanced clinical interventions.
